# Inflammatory and immune profiling in children and adolescents with attention-deficit/hyperactivity disorder (ADHD): a matched case–control study with longitudinal on/off psychostimulant assessment (ANIME)

**DOI:** 10.1186/s12888-026-08012-1

**Published:** 2026-03-27

**Authors:** Erica Fongaro, Quentin Leyrolle, Charlotte Madore, Sylvain Lehmann, Marie-Christine Picot, Sophie Laye, Diane Purper-Ouakil

**Affiliations:** 1 Department of Child and Adolescent Psychological Medicine, Montpellier University Hospital, Montpellier, Hérault, France; 2 Developmental Psychiatry/ADHD and Emotional Disorders, INSERM U 1018, CESP, Villejuif, France; 3 University Bordeaux, INRAE, Bordeaux INP, NutriNeuro, UMR 1286, Bordeaux, F-33000 France; 4 Clinical Research and Epidemiology Unit, DIM (Medical Information Department), Montpellier University Hospital, Montpellier, France; 5 LBPC-PPC, Université de Montpellier, INM INSERM, IRMB CHU de Montpellier, Montpellier, France

**Keywords:** Inflammation, Nutrition, ADHD, Polyphenols, Psychostimulants

## Abstract

**Background:**

Attention-Deficit/Hyperactivity Disorder (ADHD) is a prevalent neurodevelopmental condition with high societal impact. While psychostimulants like methylphenidate (MPH) are first-line treatments, 10–20% of patients show insufficient efficacy, and many experience side effects such as sleep and appetite disturbances. Emerging evidence suggests that neuroinflammation and oxidative stress contribute to ADHD pathophysiology. Animal studies indicate that MPH may induce pro-inflammatory processes, but this has yet to be characterized in humans.

**Methods:**

The ANIME study (ADHD Nutritional, Inflammatory, and Metabolic Endophenotypes) employs a combined case-control and prospective cohort design. A case-control study will compare the immune, inflammatory, and nutritional profiles of 40 children/adolescents with ADHD against 40 age- (± 2 years) and sex-matched typically developing controls. The ADHD group will have a follow-up visit to assess the impact of psychostimulant treatment). The primary outcome is the comparison of the immune and inflammatory markers’ levels, particularly IL-6 levels, at baseline. Secondary outcomes include deep immunophenotyping via spectral flow cytometry (Cytek Aurora) and high-sensitivity multiplex proteomics (NULISA), alongside assessments of polyphenol bioavailability, diet quality (KIDMED), and clinical symptoms.

**Discussion:**

ANIME will provide an in-depth characterization of the “inflammatory biotype” in ADHD, specifically examining how psychostimulants interact with pre-existing immune states. By integrating metabolic, nutritional, and clinical data, the study aims to identify biomarkers for treatment response and tolerability, ultimately informing personalized care and future trials of antioxidant-rich adjunct therapies like polyphenols.

**Trial registration:**

Clinicaltrials.gov. Number: NCT07417878 (https://clinicaltrials.gov/study/NCT07417878). Registered on the 13th of Febraury, 2026.

## Background

Attention deficit hyperactivity disorder (ADHD) is a common neurodevelopmental disorder (5%) with a high societal impact [1].

The pathophysiological processes underlying ADHD are likely to affect different types of brain cells. Dopaminergic neurotransmission is known to be impaired in ADHD [2]. However, recent studies have revealed that a third of the genetic risk factors for ADHD are associated with the neurodevelopmental functions of glial cells [3].

A recent animal study showed that interleukin (IL)-4, a type 2 cytokine, alters microglia function during development, leading to ADHD-like behavior [4]. Spontaneously hypertensive rats, a common animal model of ADHD, have altered MG [5]. GWAS pan-genomic studies of ADHD have revealed 12 gene loci expressed in glial cells, including microglia and macrophages [6]. The functions of MG and non-parenchymal BAMs, recognized as playing a key role in neurodevelopment, central nervous system homeostasis and neurological disease [7], may be pathophysiological pathways in ADHD [8]. A recent study on ADHD rat models showed an improvement in behavioral disorders following ingestion of prepared rehmannia root, known for its anti-inflammatory properties [4].

We are therefore in a unique position to explore the role of inflammation in the pathophysiology of ADHD.

Psychostimulants (methylphenidate, dexamphetamine, lisdexamphetamine) are the first-line drug treatments for ADHD. They enhance dopaminergic and noradrenergic transmission and demonstrate a favourable risk–benefit ratio, with well-established efficacy for inattention and hyperactivity/impulsivity [9]. However, treatment response is incomplete: 10–20% of patients show insufficient efficacy, and 20–30% experience poor tolerability [1, 10].

Emerging evidence suggests that inflammation may contribute to this variability. Preclinical studies report that methylphenidate can induce pro-apoptotic and pro-inflammatory processes in the brain [11–13]. In parallel, small clinical studies have identified altered immune profiles in children with ADHD, including increased neutrophils and NK cells [14, 15], suggesting that inflammatory alterations may arise early in the disorder. Given the tightly regulated and bidirectional interaction between the brain and immune systems [16], it is plausible that psychostimulant exposure interacts with a pre-existing inflammatory state. Yet, no study has directly examined immune and inflammatory markers in ADHD according to psychostimulant treatment status.

Dietary factors may further modulate this inflammatory dimension. Nutrients with anti-inflammatory properties, particularly polyphenols, have shown neuroprotective and cognitive benefits in animal and human studies [17–22]. Plant-rich or Mediterranean dietary patterns are associated with reduced oxidative stress, lower systemic inflammation, and decreased ADHD prevalence [23–26]. One study has linked higher polyphenol intake to reduced ADHD rates in children [27]. However, their potential influence on treatment response and inflammatory status in ADHD remains unexplored.

This study will be the first to comprehensively assess immune and inflammatory markers in children with ADHD according to psychostimulant treatment, dietary polyphenol intake, and metabolic profile. Using high-dimensional immune profiling technologies (Cytek Aurora, NULISA), our consortium will characterize immune signatures in treated and untreated patients [28]. By integrating inflammatory, metabolic, and nutritional data with treatment outcomes, this work aims to improve patient stratification and inform future clinical trials of polyphenols as adjunct therapies [29], ultimately contributing to more personalized and accessible care [30].

### Objectives

#### Main objective

The primary objective of this study is to compare the immune and inflammatory profiles, particularly IL-6 levels, of children and adolescents with ADHD versus their age- (± 2 years) and sex-matched typically developing peers.

#### Secondary objectives

1. Compare immune-inflammatory profiles of children and adolescents with ADHD vs. age- and sex-matched typically developing peers.
2. Compare immune-inflammatory profiles according to psychostimulant treatment status (under treatment or off-treatment).
3. Explore the existence of distinct immune-inflammatory biotypes among children and adolescents with ADHD using unsupervised approaches (High Inflammatory Profile vs. Low Inflammatory Profile).
4. Investigate associations between immune-inflammatory profiles and:
  - ADHD symptoms,
  - gastrointestinal (GI) symptoms,
  - treatment tolerance and efficacy.
  - sleep.
  - experienced adverse life events.

5. Compare the nutritional profiles of children and adolescents with ADHD with those of their typically developing peers.
6. Analyze the links between nutritional profiles, immuno-inflammatory profiles and clinical symptoms (ADHD and GI).
7. Evaluate polyphenol bioavailability and its association with.
  - immuno-inflammatory profiles,
  - tolerance and efficacy of psychostimulant treatment.
  - sleep.

8. Assess creativity during the treated and untreated period of children and adolescents with ADHD relative to typically developing peers.
9. Compare childhood adversity between the two groups (ADHD vs. control).

## Methods

### Trial design

Our study design is tailored to address the research questions while minimizing intervention in children and adolescents.

The combination of case-control, cross-sectional and longitudinal cohort (maximum 1-year follow-up) approaches reduces participant attrition and enhances study feasibility.

Controls will be recruited from the case’s natural environment and matched by age and sex, improving group comparability and better controlling for biases. Furthermore, primary and secondary outcome measures are collected prospectively, minimizing recall bias. Our primary outcome is a biological variable, measured blind to clinical data in a laboratory separate from the clinical inclusion site, which will limit differential misclassification between participants with ADHD and controls. This design will also enable evaluation of other ADHD-related exposures such as nutritional profile.

The clinical cohort with repeated measurements allows assessment of psychostimulant treatment effects (efficacy and tolerability) on peripheral inflammatory and immune profiles in ADHD children/adolescents. Tracking immune/inflammatory profile changes through treatment sequences (on/off) will capture treatment effect dynamics on targeted biomarkers. In this design, each participant serves as their own control, ensuring better result comparability and improved feasibility through smaller required sample sizes. Short follow-up duration and non-interference with routine treatment limits potential attrition and study dropouts. Moreover, this study type provides statistical advantages through potential use of mixed models accounting for within-subject variability and data dependence, if model conditions are met.

Finally, a cross-sectional design is optimal for examining relationships between polyphenol nutritional profile, polyphenol bioavailability in ADHD children/adolescents, and their inflammatory and treatment response profiles.

These three study designs address the long-term clinical objective of better stratifying patients according to their profiles for more individualized care. An adjuvant treatment to psychostimulant therapy based on polyphenols appears to be a promising avenue for some individuals with ADHD.

### Study setting

This study will take place in outpatient psychiatric settings, targeting patients receiving routine care for ADHD, including pharmacological treatment and regular psychiatric follow-up and healthy control individuals matched by age (± 2 years) and sex. Recruitment will begin in April, 2026 and is expected to be completed by October 2027.

### Eligibility criteria

#### Inclusion criteria

Group ADHD:

- Children or adolescents aged 7 to 17 years.
- Meeting DSM-5 criteria for ADHD (assessed using the K-SADS).
- Currently treated with psychostimulants.
- Stabilized on psychostimulant treatment for at least one month.
- Signed parental consent obtained.
- Assent obtained from the participating minor.
- Affiliated with a national health insurance scheme.

Controls:

- Children or adolescents aged 7 to 17 years without a diagnosis of a mental disorder.
- Matched to cases by age and sex.
- Signed parental consent obtained.
- Assent obtained from the participating minor.
- Affiliated with a national health insurance scheme.

#### Non-inclusion criteria

Group ADHD:

- Progressive neurological disorder, intellectual developmental disorder, or clinically significant suicide risk.
- Immunological or chronic inflammatory disease.
- Inability to comply with treatment-free periods of at least two weeks.
- Absence of parental consent.
- Absence of assent from the participating minor.
- Participation in another research study with an ongoing exclusion period.

### Controls

- Immunological or chronic inflammatory disease.
- Absence of parental consent.
- Absence of assent from the participating minor.
- Participation in another research study with an ongoing exclusion period.

#### Exit criteria

Participation in the study is voluntary. Participants have the right to withdraw their consent at any time for any reason without any negative consequences.

Early termination criteria.

○ Withdrawal of consent.

○ Intercurrent illness or condition interfering with continued study participation.

○ Investigator’s decision with explicit justification.

For each early termination, the investigator must document the date and reason in both the medical record and case report form (CRF). If the termination reason is an adverse event (AE), the specific event must be reported in the CRF. The investigator will ensure proper documentation of its outcome. Safety data from participants who terminate early or are lost to follow-up will be analyzed.

### Criteria for discontinuing or modifying the allocated interventions

The study will normally conclude as planned in the protocol.

However, certain circumstances may lead to temporary suspension or early termination:

- Significant Good Clinical Practice (GCP) violations compromising the study’s primary objectives or participant safety.
- Occurrence of local or global events (e.g., COVID-19 pandemic) impacting study participant safety monitoring.
- Explicit request by a regulatory authority to stop treatment for individual participants or all study subjects when continued participation becomes inappropriate based on efficacy evidence.

The sponsor may decide to discontinue the study at any time for the following reasons:

- insufficient recruitment rates.
- data quality issues.
- unforeseen site conditions requiring trial interruption.
- or emerging scientific/ethical concerns requiring study termination.

All discontinuation reasons must be thoroughly documented.

If an investigator or the Independent Safety Committee (CSI) has ethical concerns about the study conduct, the coordinating investigator must be immediately notified.

In case of premature termination by the sponsor or coordinating investigator, the Competent Authority and Committee for the Protection of Persons (CPP) must be informed within timelines specified by national and European regulations.

The Competent Authority may suspend or prohibit the study if authorization conditions are not met or if concerns arise regarding study safety or scientific validity.

### Explanation for the choice of comparators

The comparator group consists of typically developing children and adolescents, matched to the ADHD group by age (± 2 years) and sex with no history of neurodevelopmental, psychiatric, or chronic inflammatory disorders. This choice allows for the identification of immune and inflammatory alterations that are specific to ADHD, while controlling for potential confounders such as age, sex, and baseline inflammation. Excluding individuals with mental health conditions or inflammatory diseases ensures that the control group represents a biologically and clinically healthy population. This enhances the validity of between-group comparisons and supports the identification of ADHD-specific immuno-inflammatory signatures.

We chose a 6-month evaluation period to assess both the effects of the psychostimulants, during the no treatment period (a minimum period of 2 weeks) and the treatment period. This timeframe is long enough to capture meaningful clinical outcomes while remaining feasible.

### Relevant concomitant care permitted or prohibited during the trial

Participants in the ADHD group will be on stable psychostimulant treatment for at least one month prior to inclusion. Psychostimulant treatment is prescribed as part of routine clinical care and is not modified by the study.

Given the study’s focus on inflammatory markers, any concomitant use of anti-inflammatory medications (e.g., NSAIDs, corticosteroids) must be reported to the investigator. While the study does not restrict such prescriptions, their use will be documented and monitored throughout the study period to control for potential confounding effects on immune/inflammatory biomarkers. Participants must not take dietary supplements rich in polyphenols during the study period.

### Primary endpoints

Compare IL-6 levels in children and adolescents with ADHD to those of age- (± 2 years), and sex-matched typically developing peers.

### Secondary endpoints

1. Compare immuno-inflammatory markers’ levels in children/adolescents with ADHD versus age- and sex-matched typically developing peers.
2. Compare biomarkers according to psychostimulant treatment status (treated vs. untreated).
3. Explore the existence of distinct immuno-inflammatory biotypes in children/adolescents with ADHD using unsupervised approaches (High Inflammatory Profile vs. Low Inflammatory Profile; methods described in Schnorr et al., 2024 [31]).
4. Investigate associations between immuno-inflammatory profiles and:
  - ADHD symptoms (ADHD Rating Scale (ADHD-RS) [32], Clinical Global Impression scales (CGI) [33], Affective reactivity Index- Parent Version, (ARI-P) [34],
  - Gastrointestinal symptoms (Pediatric Diagnostic Questionnaire (R4DQ)) [35],
  - Treatment tolerability and efficacy (Pediatric Adverse Event Rating Scale (PAERS) [36], Visual Analogue Scale (VAS) [37].
  - Experienced adverse life events (Pediatric Adverse Event Rating Scale, ACE-IQ) [39].

5. Compare nutritional profiles (assessed via Mediterranean Diet Quality Index for children and adolescents, KID-MED) [25] of children/adolescents with ADHD to those of typically developing peers.
6. Analyze relationships between nutritional profiles (KIDMED) [25], immuno-inflammatory profiles and clinical symptoms (ADHD-RS, CGI, ARI-P, R4DQ, PAERS, VAS) [32–37].
7. Evaluate polyphenol bioavailability (calculated using the food diary and Phenol-Explorer (http://phenolexplorer.eu/)) and its association with:
  - ADHD symptoms (ADHD-RS, CGI) [32, 33],
  - Irritability (ARI-P) [34],
  - Immuno-inflammatory profiles,
  - Psychostimulant treatment tolerability/efficacy (PAERS, VAS) [36, 37].

8. Assess creativity during the treated and untreated period of children and adolescents with ADHD relative to typically developing peers (divergent thinking task and remote association paradigms).
9. Compare childhood adversity (ACE-IQ) [39] between the two groups (ADHD vs. control).

### Other assessments

A detailed socio-demographic and early development questionnaire will be used to collect information about the child’s age, gender, family structure, socio-economic status, and developmental milestones, as well as previous medical or psychological interventions.

### Interventions

This clinical study includes a comprehensive set of assessments and biological analyses to characterize immune-inflammatory, nutritional, and clinical profiles in children with ADHD, compared to a group of healthy controls. Data will be collected during multiple study visits: V1, V2 for the ADHD group (V2 evaluation will be conducted either under treatment or off-treatment conditions, depending on the treatment status at the previous visit); V1 only for the control group, using both standardized questionnaires and state-of-the-art laboratory techniques. The Standard Protocol Items: Recommendations for Interventional Trials (SPIRIT) [40] is outlined in Table 1 (ADHD) and Table 2 (Controls).

### Pre-inclusion visit

The pre-inclusion visit for children with ADHD may take place following a clinical follow-up appointment. The site investigator or the attending clinician will provide the families with essential information about the research and will give them the information sheet along with the consent forms. An investigator from Montpellier will then contact the families by phone to answer any questions, verify the inclusion and exclusion criteria and plan the pre-inclusion visit.

During the pre-inclusion visit, children with ADHD will be evaluated to confirm their diagnostic status. This evaluation includes the K-SADS interview if it has not been conducted in the six months preceding the pre-inclusion.

Families of children who are controls and are interested in the study can contact the investigator, and detailed information will be provided to them. The information sheet will then be sent. A telephone pre-inclusion visit will be arranged to verify the eligibility criteria for their child.

### Inclusion visit (V1)

After the informed consent is signed and the inclusion and exclusion criteria are verified, the inclusion visit evaluation will be carried out at the outpatient clinic at the CHU Montpellier as well as collaborating clinical centers, with the evaluator, involving the children/adolescents, parents, and clinicians (duration of 1 h).

Assessments of children/adolescents:

- Blood sample: 1 tube (7 mL) EDTA for plasma and 1 tube CPTBD (7 mL) heparinized for peripheral blood cells (PBMCs). For children weighing more than 30 kg, two 7 mL EDTA tubes will be collected specifically for research purposes.
- Creativity tasks (divergent thinking and associative combinations).
- ACE-IQ.

Assessments of parents:

- Sociodemographic and early developmental questionnaire.
- Current medication status of the child.
- P-ARI.
- R4PDQ.
- KIDMED.
- Food diary.
- SDSC.

Clinician’s assessment:

- CGI-S.

Exclusively for the ADHD group (treated and untreated):

- ADHD-RS)
- VAS.
- PAERS.

### Follow-up evaluations (V2: Exclusively for the ADHD group)

Follow-up is planned exclusively for the ADHD group. The follow-up evaluation will be conducted either under treatment or off-treatment conditions, depending on the treatment status at the previous visit (duration of 1 h). Evaluations off-treatment must be at least 2 weeks of no psychostimulants. Annual treatment breaks are recommended to reassess the need to continue the prescription (NICE 2018 [41]). Participation in the study will not interfere with the medication prescription. The off-treatment evaluation will take place in the weeks following the return of children/adolescents to school to avoid confounding factors related to holidays and the harmful effects of extended off-treatment periods.

Assessments of children/adolescents:

- Blood sample: 1 tube (7 mL) EDTA for plasma and 1 tube CPTBD (7 mL) heparinized for peripheral blood cells (PBMCs). For children weighing more than 30 kg, two 7 mL EDTA tubes will be collected specifically for research purposes.
- Creativity tasks (divergent thinking and associative combinations).
- VAS.
- ACE-IQ.

Assessments of parents:

- Sociodemographic and early developmental questionnaire.
- Current medication status of the child.
- P-ARI.
- R4PDQ.
- KIDMED.
- ADHD-RS.
- PAERS.
- Food diary.
- SDSC.

Clinician’s assessment:

- CGI-I.

**Table 1:** Schedule of enrollment, interventions and assessments of primary and secondary assessments for ADHD group; V = visit

Visit (V)	Pre-inclusion V0 Screening	Inclusion V1 (under treatment or off-treatment) Between 7 days from V0 and 6 months after V0	Follow-up V2 (under treatment or off-treatment) Between 1 and 6 months after V1
Study Presentation	X		
Information	X		
Consent		X	
Inclusion/Exclusion Criteria	X		
Sociodemographic Data		X	
Stimulant Treatment Status		X	X
K-SADS (if more than 6 months old or never conducted)	(X)		
ADHD-RS		X	X
R4PDQ		X	X
CGI-I		X	
CGI-S			X
ARI-P		X	X
KIDMED		X	X
VAS		X	X
PAERS		X	X
ACE-IQ		X	X
SDSC		X	X
Creativity’s tests		X	X
Food diary		X	X
Blood Sample		X	X

**Table 2:** Schedule of enrollment, interventions and assesments of primary and secondary assessments for control group; V = visit

Visit (V)	Pre-inclusion V0	Inclusion V1 Between 7 days V0 and 6 months after V0
Study Presentation	X	
Information	X	
Consent		X
Inclusion/Exclusion Criteria	X	
Sociodemographic Data		X
Stimulant Treatment Status		X
Questionnaires and Clinical Evaluations		
ARI-P		X
R4PDQ		X
KIDMED		X
ACE-IQ		X
Creativity’s tests		X
Food diary		X
SDSC		X
Biomarkers		
Blood Sample		X

### Outcomes

**Primary outcome measure**:

- Biomarkers levels, in particular the circulating IL-6 levels, will be compared at V1 between children with ADHD and typically developing controls.

**Secondary outcome measures**:

- Circulating inflammatory markers will be quantified using a targeted immune-metabolic biomarker approach. Analyses will be conducted at V1 comparing children with ADHD and controls, and longitudinally within the ADHD group between V1 and V2.
- In addition, peripheral immune and inflammatory profiles associated with ADHD will be characterized using spectral flow cytometric analysis of peripheral blood mononuclear cells (PBMCs). The team of Prof. Purper-Ouakil (P1) will collect whole blood, and the CHU Montpellier Biological Resource Center (LBPC thematic CRB, P3) will isolate PBMCs using dedicated collection tubes. Approximately 20 × 10⁶ PBMCs will be cryopreserved immediately and shipped to National Research Institute for Agriculture, Food and Environment (INRAE) Bordeaux (P2). The INRAE team will perform peripheral immune phenotyping using spectral flow cytometry (Cytek Aurora) with a predefined panel of markers targeting major immune cell populations. The immunophenotyping panel includes 20 directly fluorophore-conjugated markers to identify specific subpopulations (e.g., CD4, CD8, CD16) and activation markers (e.g., CD69, CD86), depending on cell type representation [42]. Analyses will be performed at V1 between ADHD and control groups, and longitudinally within the ADHD group between V1 and V2.
- To further explore novel inflammatory biomarkers in ADHD, advanced proteomic profiling will be conducted using the NULISA (Nucleic Acid Linked Immuno-Sandwich Assay) platform (P3). This technology, currently available in a limited number of laboratories, employs a dual capture-and-release immunocomplex mechanism that significantly improves signal-to-noise ratio and enables attomolar sensitivity (10⁻¹⁸ mol). This high sensitivity is achieved through next-generation sequencing readout, providing a dynamic range of up to 12 orders of magnitude and enabling high-throughput multiplex analyses. The panel covers 120 targets, key pathways involved in neurodegeneration, neuroinflammation, synaptic dysfunction, vascular injury, and metabolic dysregulation [43]. Based on the NULISAseq Neuro 120 panel, the biomarkers (See Glossary) can be grouped into the following major biological categories: Inflammation and immune response markers, including cytokines and chemokines (IL-1β, IL-6, IL-10, IL-18, TNF-α, IFN-γ, CCL2, CCL3, CCL4, CCL5, CXCL8, CXCL10, CX3CL1), immune receptors and signaling mediators (TREM2, CD33, ICAM1, VCAM1), and acute phase/inflammatory proteins (CRP, SAA1, CHI3L1, S100B). Neurodegeneration and neuronal structural/synaptic proteins, including axonal injury markers (NEFL/NfL, NEFH, UCHL1), synaptic proteins (SNAP25, NRGN, NPTX2, VGF, DLG4), tau and amyloid pathway markers (tTau, pTau181, pTau217, Aβ38, Aβ40, Aβ42, BACE1, PSEN1), and aggregation-related proteins (SNCA, TARDBP/TDP-43, SOD1, SQSTM1). Glial activation markers, including GFAP, AQP4, CHI3L1, TREM2, and CST3, reflecting astrocytic and microglial responses. Metabolic and mitochondrial markers, including ApoA1, ApoE, LDLR, IGF1R, IGFBP7, FGF21, GDF15, and oxidative stress–related proteins such as PRDX6 and HMOX1. Growth factors and vascular/angiogenesis markers, including VEGF-A, VEGF-D, PGF, FLT1, KDR, FGF2, PDGFRB, and TEK, capturing blood–brain barrier and vascular integrity pathways. Lysosomal and proteostasis pathway components, including PRKN, GBA-related proteins, and ubiquitin–proteasome system markers. *Neurotrophic and neuronal signaling factors*, including BDNF, NGF, NTRK2, and related signaling mediators. Extracellular matrix and adhesion molecules, including L1CAM, NCAM1, and selected matrix-associated proteins. State-of-the-art methodologies from INRAE Bordeaux (Cytek Aurora spectral cytometer for deep PBMC phenotyping) and Institute for Regenerative Medicine and Biotherapy (IRMB) CHU Montpellier (NULISA proteomics platform) will be combined. The integration of spectral flow cytometry and NULISA proteomics will allow, for the first time, an in-depth characterization of immune profiles in children with ADHD, with or without psychostimulant treatment. Analyses will be conducted at V1 between ADHD and control groups and longitudinally within the ADHD group between V1 and V2.
- In the clinical group, tolerability to psychostimulant treatment will be assessed using the PAERS [36], completed by the clinician, combined with spontaneous patient feedback using a VAS. The VAS consists of a horizontal line anchored by “no pain” and “worst imaginable pain.” The PAERS is a parent-report questionnaire assessing frequency and severity of known psychostimulant-related adverse events (neuropsychiatric, cardiovascular, gastrointestinal, etc.). Each adverse event is rated according to its attribution to medication (0 = no; 1 = yes; 2 = other medication; 3 = drug–drug interaction) and severity (1 = no functional impairment; 2 = mild; 3 = moderate; 4 = severe impairment in daily functioning). This assessment will be performed at V1 and V2.
- Nutritional profiles will be evaluated using the KIDMED [25], a 16-item questionnaire (12 positive, 4 negative items). It will be administered by a qualified health professional to parents at V1 and V2 for ADHD cases and at V1 for controls. Negative items score − 1 and positive items + 1. Total scores are categorized as follows: ≥8 indicates optimal adherence; 4–7 indicates partial adherence requiring dietary improvement; ≤3 indicates very low adherence to the Mediterranean diet.
- Gastrointestinal symptoms will be assessed using the R4PDQ [35], a parent-completed instrument for functional gastrointestinal disorders. It will be administered at V1 and V2 in cases and at V1 in controls.
- ADHD symptom severity will be assessed in the clinical group using the CGI-Severity and CGI-Improvement [33], standardized ADHD symptom scales [32], and the parent-rated ADHD-RS. These measures will be administered at V1 and V2.
- Irritability will be evaluated using the parent-reported ARI [34] at V1 and V2 in cases and at V1 in controls. The ARI assesses frequency and intensity of emotional reactivity, particularly irritability, including ease of anger, duration of outbursts, and difficulty returning to baseline. Items are rated from “never” to “very often.”
- Sleep–wake patterns and sleep disturbances will be assessed using the SDSC, administered at V1 in controls and at V1 and V2 in cases [38].
- Average daily polyphenol intake will be estimated using a 3-day food diary completed during the week preceding blood sampling (two weekdays and one weekend day). Intake will be calculated using the tool developed by Hinojosa-Nogueira et al. (2021) [44], based on the Phenol-Explorer database, which compiles over 35,000 values covering 500 polyphenols across more than 400 foods. This assessment will be performed twice during the study: once under regular treatment conditions and once during the annual treatment interruption. Intakes will be expressed in mg/day. Analyses will be conducted at V1 and V2 in cases and at V1 in controls.
- Polyphenol bioavailability will be assessed using a targeted plasma metabolomics approach. Polyphenols undergo extensive intestinal and hepatic conjugation (methylation, sulfation, glucuronidation) [45] ; therefore, circulating metabolites differ from dietary forms. Ultra-high-performance tandem mass spectrometry (UHP-MS/MS) [19] will be used to quantify polyphenols and their metabolites in plasma samples (P2). This approach has previously demonstrated associations between polyphenol metabolites and cognitive performance in older adults [19]. Analyses will be conducted at V1 and V2 in cases and at V1 in controls.
- Additional biomarkers, including oxidative stress markers, may be investigated through biobank-based ancillary studies.
- The ACE-IQ, validated in French [39], assesses 29 items across 13 categories of childhood adversity. It captures three dimensions: childhood maltreatment, household dysfunction, and external violence exposure. Scores range from 0 to 13 per dimension. The instrument demonstrates good psychometric validity (Cronbach’s alpha > 0.80; intra-category correlations 0.61–0.78; established test–retest reliability). It will be administered at V1 in controls and at V1 and V2 in cases [38].
- Creativity tasks: Divergent thinking will be assessed using the Alternate Uses Task, in which participants generate as many unusual uses as possible for common objects (e.g., pen, paperclip). Outcomes include fluency (number of ideas) and originality (statistical infrequency of responses) [46, 47]. Associative combination ability will be assessed using a remote association paradigm, in which participants identify a common associative link between three seemingly unrelated words (e.g., “bread,” “culture,” “grass” → “wheat”) [48, 49]. Tasks will be administered at V1 in controls and at V1 and V2 in cases, using different stimuli at each visit.
- For the ADHD group, the Kiddie Schedule for Affective Disorders and Schizophrenia (K-SADS), a semi-structured diagnostic interview aligned with DSM-5 criteria, will be administered at V1 if the most recent evaluation is older than six months. The validated French version will be used.

### Recruitment

#### Feasibility

ANIME brings together a unique consortium of teams with highly complementary expertise in the clinical evaluation and treatment of ADHD (P1 - Equipe Pr Purper-Ouakil - CHU Montpellier) and cellular immunophenotyping through the identification of the proteomic and transcriptomic landscape of peripheral and cerebral immune and neuro-immune cells and nutritional status (P2 - Equipe Dr Laye - INRAE Bordeaux, P3 - Equipe Pr Lehmann - IRMB CHU Montpellier). We will be using cutting-edge methodologies developed in the P2 (Cytek Aurora spectral cytometer for in-depth phenotyping of PBMCs) and P3 (Nulisa proteomic, which measures a wide range of inflammatory factors [45]), laboratories. The combined use of flow cytometry and NULISA will make it possible for the first time to describe in depth the immune profiles of children with ADHD with or without psychostimulants.

### Practical recruitment procedures

#### Recruitment of individuals with ADHD

Children and adolescents aged 7 to 17 years with a diagnosis of ADHD will be systematically recruited from outpatient clinic at Montpellier University Hospital (CHU Montpellier) during the study period, as well as through collaborating clinical centers. Inclusion in the study will be systematically offered to all patients consulting during the study period who meet the inclusion criteria. An email presenting the study will be sent to parents/patients from the active file who are not consulting during the study period but meet the inclusion criteria. If the patients/parents show interest in the study, a pre-inclusion visit will be scheduled.

#### Recruitment of healthy controls

Controls, matched by age (± 2 years), and sex with the ADHD group, will be recruited from the friends, acquaintances, or social circles of children/adolescents with ADHD, and from children/adolescents in the local population through an announcement published in the press, on the CHU intranet, and via information leaflets and posters. The parents from the ADHD group can provide information leaflets about the study to their acquaintances. Families interested in the study can contact the investigator, and detailed information will be provided. The information sheet will then be given to them after verifying their child’s eligibility criteria, and consent must be signed before participation in the study. Children related to the participants from the ADHD group cannot be controls for the study due to their shared genetic background.

Announcements authorized by the CPP will be used to facilitate the recruitment of subjects (letters, posters, University Hospital’s intranet, local press). The investigator will present the study to potential participants. He will explain the nature of the study and its procedure in such a way that the subject will understand their rights and responsibilities, as well as the potential risks and benefits.

The investigator will ensure that potential participants are eligible according to the eligibility criteria.

#### Practical procedures for obtaining consent

Informed consent as well as the assent of children and adolescents will be obtained before any study-specific procedures are carried out. It will be specified to the participants that their participation is entirely voluntary and that they can withdraw from the study at any time.

The investigator will make efforts to obtain consent from both parents. If consent is obtained from only one parent, this situation must be justified by the investigator. Participation in the study is still possible with consent from only one parent. In accordance with GCP, before obtaining the informed consent, the subject and the parents/legal representatives of the subject will be informed of all aspects of the study that are important and relevant to make an informed decision about participation. They will also be given sufficient time to reflect and the opportunity to ask any questions they may have. If they wish, they may be assisted by a trusted person.

The informed consent process will be documented in the participant’s medical file.

### Implementation

The study will be coordinated by the Montpellier team, in particular Prof. D. Purper-Ouakil.

The promoter, Montpellier University Hospital, is responsible for the administrative and financial management of the study, the quality of the trial, and the choice of investigators and sites.

The investigator is responsible for recruiting patients, providing information to the patient (and/or legal representative, guardian/trusted person) and obtaining informed consent. The investigator is responsible for the quality of the trial, and for compliance with GCP and the protocol for the duration of the trial. The investigator is responsible for informing subjects and obtaining their informed consent.

### Data collection methods

Each assessment will be collected for each participant in accordance with the study data collection form by the investigator(s) or the clinical study technician(s) in charge of the study.

### Data management

The individual data required to analyse the study must be:

- entered into the electronic-CRF (e-CRF) as they are obtained, whether they are clinical or para-clinical data.
- anonymised by the investigator.
- authenticated by an electronic signature from the investigator.
- all data are entered, which means that missing data must be justified.

Initially, the data entered into the e-CRF is checked and validated by the Clinical Research Monitor on the basis of the source documents.

Secondly, the data manager performs additional computerised consistency tests based on the presence of non-standard, missing, aberrant or inconsistent data, which are performed regularly during patient recruitment and follow-up. All consistency tests are defined in advance in a set of specifications proposed by the data manager and validated by the investigator, the statistician, the CRA monitor and the study TEC. Each inconsistency identified in the e-CRF (‘Query’) is the subject of a request for correction or justification to the investigator (response to the ‘Queries’). The investigator undertakes to make himself available to the members of the research team and to provide them with any additional information required to resolve these errors. This information is recorded in the study database.

Once the study has been completed, i.e. the data required by the protocol and any additional data entered (self-questionnaires), and the data monitored and validated, the study database is frozen.

The Clinical Research and Epidemiology Unit (URCE) at Montpellier University Hospital is responsible for maintaining the database. The data is stored in ASCII format.

### Identification of the subject

To ensure participant anonymity, each subject will be identified by a unique identification number consisting of the first letter of their last name, first letter of their first name, gender, and year of birth. A subject identification list will be maintained in the investigator’s file. The investigator will guarantee proper pseudonymization for each study participant. All participant data will be recorded in a standardized case report form completed by the investigator, co-investigator, or assigned Clinical Research Technician (CRT) or CRA.

### Observation booklet

Pseudonymized study data are entered via an e-CRF enabling real-time data quality control. The e-CRF will be developed using Ennov Clinical software to ensure data entry quality.

This software complies with FDA guidance for computerized systems in clinical trials (“Guidance for Computerized Systems Used in Clinical Trials”), electronic signature requirements (21 CFR Part 11), and other international standards (CDISC, ICH, GCP 2001/20/EC, etc.). System access requires unique usernames and passwords specific to each authorized user.

The system includes comprehensive audit trail functionality for complete tracking and supervision of all user actions. Encrypted data are transmitted to the data manager through secure internet connections. The e-CRF is designed to capture all relevant medical information for enrolled patients.

### Data processing

The individual data needed to analyse the study must be:

- entered into e-CRF as and when they are obtained, whether they are clinical or para-clinical data.
- pseudoanonymised by the investigator.
- authenticated by an electronic signature of the investigator.
- This means that any missing data must be justified.

Initially, the data entered in e-CRF is checked and validated by the CRA monitor on the basis of the source documents.

Secondly, the data manager performs additional computerised consistency tests based on the presence of non-standard, missing, aberrant or inconsistent data, which are performed regularly during patient recruitment and follow-up. All consistency tests are defined in advance in a set of specifications proposed by the data manager and validated by the investigator, the statistician, the sponsor CRA and the study CRT. Each inconsistency identified in the e-CRF (“Query”) is the subject of a request for correction or justification to the investigator (response to the “Queries”). The investigator undertakes to make himself available to the members of the research team and to provide them with any additional information required to resolve these errors. This information is recorded in the study database.

Once the study has been completed, i.e. the data required by the protocol and any additional data entered (self-questionnaires), and the data monitored and validated, the study database is frozen.

Maintenance of the database is the responsibility of the Clinical Research and Epidemiology Unit (URCE) of the SSP in Montpellier.

### Data storage

Trial closure, including center shutdowns, will adhere to ICH Good Clinical Practice guidelines.

Research documents (regulatory files, source documents, case reports, consent forms) will be stored securely at the site during the study and archived per sponsor directives.

The investigator remains responsible for these documents throughout the regulatory retention period.

No relocation or destruction may occur without sponsor approval. Upon expiration of the retention period, the sponsor will authorize disposal. All data, documents, and reports remain subject to audit or inspection.

### Database freeze

Upon study completion—including collection of all protocol-required data and any supplementary data (e.g., self-questionnaires)—and after monitoring and validation, the study database will be locked. The database is frozen in accordance with the URCE (Clinical Research and Epidemiology Unit) procedures. The freeze is documented by a database freeze certificate and occurs only after all data have been verified and all corrections requested from the investigators have been completed.

### Statistical methods

The analyses will be carried out using R software version 4.4.0 after database lock and approval of the Statistical Analysis Plan by the Clinical Research and Epidemiology Unit at the University Hospital of Montpellier.

Any modifications made to the analysis plan will be defined by the methodologist in collaboration with the statistician and the principal investigator.

### Sample size

To estimate the sample size for the case-control study based on the primary outcomes, we used the results of Wang et al. [50], who compared cytokine levels between 41 patients with ADHD and 39 controls. A sample size of 36 participants in each group provides 90% power to detect a mean difference of -5.5 for IL-6 (i.e., a mean of 4.5 in group 1 [µ₁] and 10 in group 2 [µ₂]), assuming a common standard deviation of 7, using a two-sample t-test with a two-sided significance level of 5% (software: NQuery). This sample size was increased by 10% to account for unusable data, resulting in the inclusion of 40 participants per group. This is a case-control study involving children and adolescents with ADHD and age- (± 2 years), and sex-matched controls. The NSN is 40 per group.

### Analysis population

All enrolled participants will be included in the analysis.

#### Statistical analysis plan

A descriptive analysis of all collected variables will be performed by group (ADHD and Controls), including demographic characteristics (age, sex, socioeconomic status) and nutritional status (dietary intake and bioavailability of polyphenols). The quality of matching will be assessed by examining the comparability of individuals with ADHD and controls for matching factors.

Clinical characteristics of participants at baseline and throughout follow-up (ADHD symptoms, adherence and tolerance to psychostimulant treatment, gastrointestinal symptoms) will also be described at each time point. Means, standard deviations, medians, and quartiles will be reported for continuous variables, and frequencies and percentages for categorical variables. The proportion of missing data will be reported for each variable.

For the case-control study, a comparative analysis of sociodemographic characteristics, nutritional status, and the main study factors (e.g., cytokine levels) between the clinical group and the control group will be performed. For categorical variables, univariate odds ratios and their 95% confidence intervals will be reported. Statistical significance will be assessed using a chi-square test or Fisher’s exact test when the conditions for the chi-square test are not met. For continuous variables, mean comparisons will be conducted using appropriate statistical tests.

A multivariate analysis will be carried out using a conditional logistic regression model to identify potential risk factors for ADHD, accounting for confounding variables. Factors with a univariate analysis p-value of up to 0.20 will be included in the model. The final multivariate model will be selected using a stepwise procedure based on the likelihood ratio test. Results from the logistic regression will be presented as odds ratios with their 95% confidence intervals.

In the ADHD group, an analysis of ADHD symptoms (score) and GI symptoms (score) between under treatment or off-treatment periods will be performed using a mixed model for continuous outcome variables and a mixed logistic model for binary data. The explanatory variables will be nutritional status and inflammatory status, adjusted for confounding and matching factors. For pairwise comparison tests conducted using paired t-tests or Wilcoxon signed-rank tests, corrections for multiple testing will be applied.

We will examine the effect of stimulant treatment on ADHD symptoms, GI symptoms, nutritional status, sleep and the inflammatory score by comparing these variables at different time points.

Additionally, the relationships between clinical ADHD symptoms, treatment response and tolerance, sleep, nutritional status, and inflammatory status will also be analyzed.

#### Descriptive analysis and comparability of groups

The number of patients approached for the study and the number of subjects included in each group will be reported on the flow chart. Premature discontinuations (lost to follow-up, study withdrawals) and their reasons will be reported. Protocol deviations will be defined as major or minor by the Scientific Committee at the end of a blinded data review, during which deviations and missing or aberrant data will be fully described. A descriptive analysis of the initial characteristics of each group will be carried out. For qualitative variables, this description will include the number of participants and the frequency of the different modalities. For quantitative variables, the description will include the number of participants, the mean, the standard deviation, the median and the extreme values (minimum and maximum). To ensure comparability, patient demographic and baseline characteristics will be summarized by group using descriptive statistics. For continuous variables, descriptive statistics (n, mean, standard deviation, standard error, median, minimum, and maximum) will be provided. For categorical variables, patient counts and percentages will be provided. Categories for missing data will be presented if necessary. In the case of non-comparability for one or more parameters, an adjustment will be made on this/these parameter(s) for analysis of the judgment criteria. A multivariate linear regression model may be performed to account for potential confounders.

### Handling of missing data

In the presence of missing data, the mechanism for the appearance of this data will be studied for each of the variables. If the MNAR (Missing Not At Random) hypothesis is rejected, the M.C.A.R. (Missing Completely At Random) versus MAR (Missing At Random) hypothesis will be tested, by studying the relationship between the status of the variable (missing data or not) and the values taken by the covariates.

Under the hypothesis of a MAR or MCAR mechanism, i.e. that the probability of non-response for a variable is independent of the value taken by this variable, a multiple imputation method [51] will be implemented. Analyses will be carried out independently on each of the complete databases and their results will be taken into account in estimating the final parameters and their standard deviations.

Finally, the concordance of the results obtained between the complete database and the database after imputation will be studied. The main analysis will be based on the database with imputation of missing data in order to be as close as possible to the ITT population. The analyses will be carried out at the Clinical and Epidemiological Research Unit of the Montpellier University Hospital. All statistical tests will be two-sided and the significance threshold will be set at 5%. All statistical analyses will be carried out using SAS entreprise guide or R.

### Statistical analysis of clinical evaluation criteria

#### Analysis of primary endpoint

The primary outcome criterion will be circulating IL-6 levels compared at V1 (baseline) between children with ADHD and matched control children by age (± 2 years) and sex. IL-6 will be analyzed as a continuous variable. Distributional properties will be assessed and log-transformation applied if required. The primary comparison (ADHD vs. age- and sex-matched controls) will be performed using a linear mixed-effects model with group as a fixed effect and matched pair as a random intercept. Results will be reported as the estimated geometric mean ratio (ADHD/control) with 95% confidence interval.

#### Analysis of secondary endpoints

Secondary analyses will be conducted at V1 (cross-sectional comparisons) and, when applicable, longitudinally between V1 and V2 in the ADHD group. All models will be adjusted for relevant covariates (age, sex, BMI, medication status, and other potential confounders as appropriate). Biomarker distributions will be examined for normality and log-transformed when required.

1. Inflammatory markers: ADHD vs. controls
  Inflammation and immune response markers’ levels will be compared between children/adolescents with ADHD and age- and sex-matched typically developing controls at V1. A linear mixed models with matched pair as a random effect will be performed with effect sizes (Cohen’s d or standardized β) and 95% confidence intervals will be reported.
2. Inflammatory markers by psychostimulant treatment status
  Inflammation and immune response markers’ levels will be compared between treated and untreated ADHD participants under treatment of off-treatment conditions.
  Multivariable linear regression adjusting for age, sex, BMI, ADHD severity, and treatment duration. For within-subject comparisons (V1 vs. V2), linear mixed-effects models with subject as random intercept.
3. Identification of immuno-inflammatory biotypes
  Unsupervised clustering approaches will be used to explore distinct inflammatory profiles within the ADHD group:
  Standardization (z-scores) of biomarkers.
  Dimensionality reduction (e.g., PCA).
  Clustering methods (e.g., k-means, hierarchical clustering, or Gaussian mixture models).
  Model selection using silhouette index, BIC/AIC, and stability analysis.
  Clusters will be labeled descriptively (e.g., High vs. Low Inflammatory Profile) and compared on clinical variables using regression models.
4. Associations between inflammatory markers and clinical domains
  Multivariable linear regression models will assess associations between inflammatory biomarkers and: ADHD symptoms (ADHD-RS, CGI), Irritability (ARI-P), Gastrointestinal symptoms (R4DQ), Treatment tolerability (PAERS, VAS), Sleep disturbances (SDSC), Childhood adversity (ACE-IQ). Where appropriate, logistic regression will be used for categorical outcomes. Multiple testing correction (FDR or Bonferroni) will be applied.
5. Nutritional profile comparisons (KIDMED)
  KIDMED scores will be compared between ADHD and control groups using linear regression adjusted for age, sex, and socioeconomic indicators.
6. Nutrition–Inflammation–Clinical interactions
  Associations between KIDMED score and inflammatory biomarkers will be assessed using multivariable linear regression. To evaluate whether inflammation mediates the relationship between diet and clinical symptoms mediation analyses will be conducted.
7. Polyphenol bioavailability analyses
  Daily polyphenol intake (mg/day) and circulating polyphenol metabolites (UHP-MS/MS) will be analyzed through a correlations with ADHD symptoms (ADHD-RS, CGI), irritability (ARI-P), inflammatory biomarkers, and treatment tolerability. Moreover, a multivariable regression adjusting for diet quality (KIDMED), BMI, and medication status.
8. Creativity analyses
  Creativity measures (divergent thinking fluency/originality and remote association accuracy) will be compared: ADHD vs. controls at V1. Within ADHD group (under treatement vs. off-treatement) using paired mixed-effects models.
9. Childhood adversity (ACE-IQ)
  ACE-IQ total and subdimension scores will be compared between ADHD and control groups using linear regression models adjusted for age and sex.

### Demographic and baseline characteristics

The socio-demographic data collected will be date of birth (dd, month, year), ethnic origin, age, gender, socio-economic environment indicators of their parents such as schooling and level of education and professional activity.

### Oversight and monitoring

An independent safety monitor will oversee compliance with regulatory requirements and verify data collection, ensuring adherence to the study protocol. Acting as a representative of the study sponsor, the monitor will ensure that all trial procedures meet regulatory and ethical standards.

Before the inclusion of the first participant, the monitor will conduct an initial site visit to present the study protocol and procedures. Investigators will receive detailed completion guidelines for the CRFs to ensure consistency in data entry. Throughout the study, the monitor will have direct access to source documents necessary for verifying entries in the e-CRF and other protocol-related records.

To maintain data integrity and reliability, the sponsor or its representatives will implement key measures, including providing instructional materials to study sites, conducting start-up training sessions for investigators and study coordinators, and ensuring proper completion of clinical report forms and study procedures. Regular site visits will be conducted, and ongoing communication with study personnel will be maintained via email, phone, or fax. Data from Case Report Forms will be systematically reviewed, and quality control checks will be performed on the study database to ensure accuracy and consistency.

### Harms

Participation in the proposed intervention in this study presents only minimal potential risks: Typical risks associated with blood sampling, such as discomfort, bruising, and pain at the puncture site, absence of blood reflux, or anxiety (and, for the collector, the risk of blood exposure with potential contamination by hepatitis B, C, or HIV). Blood sampling will be carried out by qualified and experienced personnel. Any AE/adverse reactions/incidents related to the usual care of the patient will be declared by a health professional to the various circuits of sanitary vigilance applicable to each product or practice concerned (vigilance of the care, pharmacovigilance, hemovigilance, etc.) in conformity with the regulations in force.

### Auditing

An audit can be carried out at any time by people mandated by the sponsor and independent of the people carrying out the research. Its objective is to verify the security of participants and respect for their rights, compliance with applicable regulations and the reliability of data.

An inspection can also be carried out by a competent authority (ANSM for France or EMA as part of a European test for example).

The audit, as well as the inspection, can be applied at all stages of research, from the development of the protocol to the publication of results and the classification of data used or produced within the framework of the research.

Investigators agree to comply with the sponsor’s requirements for an audit and the competent authority for an inspection of the research.

### Ethics approval and consent to participate

The study will be conducted in compliance with current French regulations, including provisions governing research involving human participants: Law No. 2012 − 300 of March 5, 2012 concerning research involving human subjects and its implementing decrees (Decree No. 2016 − 1537 of November 16, 2016 on research involving human subjects, Decree No. 2017 − 884 of May 9, 2017 amending certain regulatory provisions concerning research involving human subjects), Bioethics laws (where applicable), Law No. 78 − 17 of January 6, 1978 (as amended) on Information Technology, Data Files and Civil Liberties, the Declaration of Helsinki, Good Clinical Practice, and current European regulations (Clinical trials of medicinal products for human use: EU 536/2014; Clinical investigations of medical devices: EU 2017/745).

This research also complies with the Regulation on the protection of natural persons with regard to the processing of personal data and on the free movement of such data, which repeals Directive 95/46/EC (General Data Protection Regulation or GDPR).

### Participant information and consent/non-opposition

Prior to conducting this research involving human participants, free and informed consent (Category 2 research under French regulations) must be obtained from the participant/legal representatives. This follows a detailed explanation by the investigator during a consultation and an adequate reflection period.

The participant/legal representative information document must include all elements specified in Law No. 2012 − 300 of March 5, 2012 (Jardé Law) governing human subject research. The language must be clear and easily understandable for both parents and participants.

### Protocol amendments

Any substantial amendment to the protocol, meaning any modification likely to significantly impact participant safety, study validity or results, quality/safety of investigational products, interpretation of supporting documents, or study conduct procedures, must be submitted as a written amendment request to the sponsor. Before implementing any substantial amendment, the sponsor must obtain a favorable opinion from the Ethics Committee (CPP) and, when applicable, authorization from the French National Agency for Medicines and Health Products Safety (ANSM).

Non-substantial amendments, defined as those without significant impact on any research aspect, are communicated to the CPP for information purposes only.

All amendments require validation by both the sponsor and all relevant study stakeholders before submission to the CPP and ANSM when applicable.

All protocol modifications must be communicated to all participating investigators, who must comply with the updated requirements.

Any amendment affecting participant management, risk/benefit assessment, or study constraints requires an updated participant information leaflet and revised consent/non-opposition form.

### Consent or assent

Informed consent and assent are obtained from the patients, respectively, before starting any trial specific procedure. All participants are advised that participation in research is entirely voluntary, and that they can withdraw their participation at any time. Families are not paid for their participation. The child’s psychiatrist or pediatrician initially presents the study. The protocol is then explained in detail by the clinical investigator before the signature of the informed consent by parents and child are collected using an online consent signature.

### Confidentiality

In accordance with the legislative provisions in force, persons with direct access to the source data will take all necessary precautions to ensure the confidentiality of information relating to the investigational medicinal products, the research, the persons involved and in particular their identity and the results obtained. These persons, in the same way as the investigators themselves, are subject to professional secrecy. During the research or at its conclusion, the data collected on the persons involved and transmitted to the sponsor by the investigators (or any other specialist) will be made anonymous. Under no circumstances must the names or addresses of the persons concerned appear in clear text.

The sponsor will ensure that each person taking part in the research has given their oral consent for access to individual data concerning them and strictly necessary for the quality control of the research.

### Ancillary and post-trial care

Participation in the clinical-trial does not interrupt previous care, assuring that post-trial care is guaranteed.

### Access to data

The investigator must undertake to allow direct access to the study’s source documents during audits or inspections. Most source documents are computerised and accessible after authorisation.

### Dissemination plans

#### Study report

Within one year after the completion or termination of the study, a final report will be prepared and signed by both the sponsor and the investigator. This report will be made available to the competent authority. The sponsor will also submit a summary of the final report to the CPP within the same one-year period following the end of the study.

#### Publication

The publication of the main results will acknowledge the sponsor, all investigators who enrolled or monitored participants, as well as the methodologists, biostatisticians, and data managers involved in the study. It will also include the vigilance officers responsible for participant safety analysis and the members of any committees established for the research. The publication will comply with international authorship and publication standards (Uniform Requirements for Manuscripts, ICMJE, April 2010).

#### Results communication

In accordance with Law No. 2002 − 303 of March 4, 2002, participants are informed, at their request, of the overall results of the research.

#### Plans to give access to the full protocol, participant level-data, and statistical code

In accordance with the law n°2002 − 303 of the 4th march 2002, participants are informed, at their request, of general results of research by the investigator. For research purposes, the investigator can provide the full protocol, participant level-data, and statistical code on request.

## Discussion

The ANIME project aims to study and compare the immune and inflammatory profiles, in particular the level of IL-6, of children and adolescents with ADHD compared with their age- and sex-matched typically developing peers. The different design of ANIME allows to bridge a critical gap in our understanding of ADHD by exploring the interplay between systemic inflammation, nutrition, and pharmacological treatment. By comparing ADHD patients to matched controls, we aim to confirm whether an “inflammatory biotype” exists in a subset of this population.

A unique strength of this protocol is the longitudinal assessment of psychostimulant effects on the human immune system. Preclinical concerns regarding the pro-inflammatory potential of methylphenidate necessitate human data to determine if such effects influence treatment efficacy or the common side effects that lead to treatment discontinuation. Furthermore, our focus on nutritional status, specifically polyphenol intake and bioavailability, addresses the growing interest in how dietary antioxidants may modulate neuroinflammation and cognitive performance.

By utilizing cutting-edge technologies like NULISA proteomics and spectral flow cytometry, this study will move beyond traditional markers to provide a high-resolution map of the ADHD immune landscape. These findings will clarify the biological heterogeneity of the disorder and help identify objective biomarkers associated with treatment response and tolerability.

## Data Availability

No datasets were generated or analysed during the current study.

## Declarations

### Protocol version

22nd of December 2025; version n°4.

### Composition of the coordinating center and trial steering committee

The coordination of the study will be carried out by the Montpellier team, specifically by Prof. D. Purper-Ouakil.

### Type of study

RESEARCH INVOLVING THE HUMAN PERSON (Jardé Law framework): Interventional research that involves only minimal risks and constraints, the list of which is set by order of the Minister in charge of health following an opinion from the Director General of the ANSM (category 2) - Order of 12 April 2018 setting the list of research mentioned in 2° of Article L.1121-1 of the CSP.

## Abbreviations

ACE-IQ: Adverse Childhood Experiences International Questionnaire
ARI-P: Affective reactivity Index- Parent Version
ADHD: Attention-Deficit/Hyperactivity Disorder
ADHD-RS: ADHD Rating Scale
ANSM: Agence Nationale de Sécurité du Médicament et des Produits de Santé (French National Agency for the Safety of Medicines and Health Products)
AMM: Marketing Authorization
ANIME: The ADHD Nutritional, Inflammatory and Metabolic Endophenotype study
BAM: Border-Associated Macrophages
CGI: Clinical Global Impression scales
CRA: Clinical Research Associate
CPP: Committee for the Protection of Persons
e-CRF: Electronic Case Report Form
GCP: Good Clinical Practice
GDPR: General Data Protection Regulation
GWAS: Genome-Wide Association Study
ICH: International Conference on Harmonization
INRAE: National Research Institute for Agriculture, Food and Environment
IRMB: Institute for Regenerative Medicine and Biotherapy
KID-MED: Mediterranean Diet Quality Index for children and adolescents
MPH: Methylphenidate
MG: Microglia
PBMCs: Peripheral Blood Mononuclear Cells
PAERS: Pediatric Adverse Event Rating Scale
R4DQ: Pediatric Diagnostic Questionnaire
SDSC: Sleep Disturbance Scale for Children
UHP MS/MS: Ultra-High-Performance Tandem Mass Spectrometry
VAS: Visual Analogue Scale

## Glossary

IL-1β: Interleukin 1 bêta.
IL-6: Interleukin 6.
IL-10: Interleukin 10.
IL-18: Interleukin 18.
TNFα: Tumor Necrosis Factor alpha.
IFNγ: Interferon gamma.
CCL2: Chimiokine CC ligand 2.
CCL3: Chimiokine CC ligand 3.
CCL4: Chimiokine CC ligand 4.
CCL5: Chimiokine CC ligand 5.
CXCL8: C-X-C Motif Chemokine Ligand 8 ou Interleukine 8.
CXCL10: C-X-C Motif Chemokine Ligand 10.
CX3CL1: Fractalkine ou C-X 3-C Motif chemokine ligand 1.
TREM2: Triggering receptor expressed on myeloid cells 2.
CD33: Cluster de différenciation.
ICAM1: InterCellular Adhesion Molecule.
VCAM1: Vascular cell adhesion protein 1.
CRP: C-reactive protein.
SAA1: Serum amyloid A1.
CHI3L1: Chitinase-3-like protein 1.
S100B: S100 calcium – binding protein B.
NEFL/NfL: Neurofilament Light chain.
NEFH: Neurofilament heavy chain.
UCHL1: Ubiquitin C-terminal hydrolase L1.
SNAP25: Synaptosome associated protein 25.
NRGN: Neurogranin.
NPTX2: Neuronal pentraxin-2.
VGF: Nerve growth factor inductible.
DLG4: Discs large homolog 4.
tTau: Total tau.
pTau181: Phosphorylated tau 181.
pTau217: Phosphorylated tau 217.
Aβ38: Amyloid beta 38.
Aβ40: Amyloid beta 40.
Aβ42: Amyloid beta 42.
BACE1: Bêta-secrétase 1.
PSEN1: Préséniline 1.
SNCA: Synuclein alpha.
TARDBP/TDP-43: TAR DNA binding protein.
SOD1: Superoxide dismutase 1.
SQSTM1: Sequestosome 1.
GFAP: Glial Fibrillary Acidic Protein.
AQP4: Aquaporin-4.
CHI3L1: Chitinase 3-like1.
TREM2: Triggering Receptor Expressed On Myeloid Cells 2.
CST3: Cystatin 3.
ApoA1: Apolipoprotein A1.
ApoE: Apolipoprotein E.
LDLR: Low- density lipoprotein receptor.
IGF1R: Insuline like growth factor 1 receptor.
IGFBP7: Insuline like growth factor binding protein 7.
FGF21: Fibroblast growth factor 21.
GDF15: Growth Differentiation Factor 15.
PRDX6: Peroxiredoxin-6.
HMOX1: Heme oxygenase-1.
VEGF-A: Vascular endothelial growth factor A.
VEGF-D: Vascular endothelial growth factor D.
PGF: Placental growth factor gene.
FLT1: Vascular endothelial growth factor receptor 1 gene.
KDR: Kinase insert domain receptor.
FGF2: Fibroblast growth factor − 2.
PDGFRB: Platelet derivated growth factor receptor alpha.
TEK: Receptor tyrosine kinase.
PRKN: Parkin encoded by the PRKB gene.
GBA-related proteins: Glucocerebrosidase.
BDNF: Brain- derived neurotrophic factor.
NGF: Nerve Growth Factor.
NTRK2: Neurotrophic receptor tyrosine kinase 2.
L1CAM: L1 cell adhesiin molecule.
NCAM1: Neural cell adhesion molecule 1.
